# Interpersonal art psychotherapy for the treatment of aggression in people with learning disabilities in secure care: a protocol for a randomised controlled feasibility study

**DOI:** 10.1186/s40814-017-0186-z

**Published:** 2017-10-10

**Authors:** Simon S. Hackett, John L. Taylor, Mark Freeston, Andrew Jahoda, Elaine McColl, Lindsay Pennington, Eileen Kaner

**Affiliations:** 1 Institute of Health & Society, Faculty of Medical Sciences, Newcastle University, The Baddiley-Clark Building, Richardson Road, Newcastle upon Tyne, NE2 4AX UK; 2 Northumberland, Tyne & Wear NHS Foundation Trust, Newcastle upon Tyne, UK; 3 Northumbria University, Newcastle upon Tyne, UK; 4 Institute of Neuroscience, Faculty of Medical Sciences, Newcastle University, Newcastle upon Tyne, UK; 5 Institute of Health and Wellbeing, Glasgow University, Glasgow, UK

**Keywords:** Feasibility study, Art psychotherapy, Art therapy, Learning disability, Secure care

## Abstract

**Background:**

Art psychotherapy has greater potential for use with adults with mild to moderate learning disabilities as it places less of a burden on verbal interaction to achieve positive therapeutic, psychological, and behavioural goals. The feasibility study objectives include testing procedures, outcomes, validated tools, recruitment and attrition rates, acceptability, and treatment fidelity for manualised interpersonal art psychotherapy.

**Methods:**

Adult males and females with mild to moderate learning disabilities will be recruited from four NHS secure hospitals. Twenty patients will be recruited and randomly assigned to one of two treatment groups: fifteen 1-h individual sessions of manualised interpersonal art psychotherapy, or a treatment as usual waiting list control group. The Modified Overt Aggression Scale will be administered to both treatment arms. Four patients will be recruited to a single-case design component of the study exploring the acceptability of an attentional condition.

**Discussion:**

This multi-site study will assist in future trial planning and inform feasibility including, procedures, treatment acceptability, therapist adherence, and estimation of samples size for a definitive RCT.

**Electronic supplementary material:**

The online version of this article (10.1186/s40814-017-0186-z) contains supplementary material, which is available to authorized users.

## Background

Art therapies, such as art psychotherapy, are currently used in a range of NHS healthcare settings. They are included in the UK National Institute for Health and Care Excellence (NICE) adult and children and young people’s guidelines for psychosis and schizophrenia and the guidelines for the treatment of depression in children and young people [1–3]. Although art therapy is included in NICE guidelines for psychosis and schizophrenia in adults, a Cochrane review of art therapy [4] (two trials, *n* = 137) concluded that the evidence of benefits and harms was inconclusive.

A systematic narrative review of 15 trials [5] of group art therapy reported lower levels of anxiety and depression, including a study of incarcerated men. The art therapy trails reviewed were targeted at symptoms of depression, anxiety, low mood, trauma, distress, reduced QoL, low coping skills, and self-esteem [5]. However, the studies were generally considered to be of low quality and the need for future high-quality research in this area was identified [5, 6]. A systematic review of art therapy studies for traumatised adults found 6 clinical evaluations (*n* = 223) but just one RCT including art therapy plus cognitive behaviour therapy for victims of sexual assault and victims with post-traumatic stress disorder (PTSD (*n* = 8)) [7]. A realist review of art therapy for depression included 16 papers and identified 8 therapeutic factors that explained how art therapy works, including self-expression, communication, understanding, and creativity [8].

A review of art therapy literature for people with learning or intellectual disabilities [9] found one RCT. Art facilitation with adults (*n* = 19) showed a difference between language comprehension and social interaction for the treatment group against the control group with an analysis of covariance of post-test with pre-test scores as covariates (*p* < .10) [10].

For people with learning disabilities, art psychotherapy uses art media as its primary mode of communication and having artistic skill and experience in art is not a prerequisite. This can offer the opportunity for expression and communication in the context of a psychological therapy to people who find it hard to express their thoughts and feelings verbally [9]. Through focusing communication around visual material there is greater potential for people with a learning disability, limited English proficiency (LEP), and/or a mental health difficulties, to develop understanding about themselves/their health or behaviour/and what they can do to enhance their well-being [11].

In offender populations a meta-synthesis review of the role of art therapies in therapeutic goals identified associations with improvements in emotional literacy, quality of life, and lower levels of arousal linked with feelings of anger [12]. Examples of ‘proof of concept’ work in adult offenders with learning disabilities have indicated positive outcomes from art psychotherapy in single-case designs. Reported improvements included reduced levels of aggression after receiving up to twenty individual sessions of treatment [13–15]. This preliminary work has led to the development of a working manual for interpersonal art psychotherapy that is being considered in this study.

Development of health technology and therapeutic interventions that support a reduction in aggressive behaviour in adults with learning disabilities has potential for clinical benefits for patients in England. Data collected as part of a national learning disability census identified a population of 3230 adults with learning disabilities being treated in hospital with a total of 1780 patients (55%) having one or more recorded incident of self-harm, accidents, physical assault, restraint, or seclusion (either ‘present’ or ‘severe’ enough risk to require hospital treatment). Within this group of patients, 5460 separate types of risk were recorded for either violence, self-injury, or damage to property [16]. An international literature review of inpatient violence and aggression [17] reported that patients treated in forensic settings are likely to be more violent than those in other settings such as psychiatric units.

Future art therapy trials could mitigate potential performance bias through being allocated to the active treatment group and assess the relative efficacy of art therapy using an attention art-based placebo control to ensure that groups are treated equally [5]. In an attempt to explore a possible comparator for a future trial, the acceptability of an attentional control condition will be tested using a single-case design [18] in parallel to the randomised waiting list control study.

In an attentional condition the level of time and attention given to the participant should match that of the treatment (structural equivalence of dose) and have a rationale for therapeutic benefit [19]. Relaxation therapy to treat symptoms of anxiety is identified as specific psychological intervention that can be considered with adults with learning disability and mental health problems [20].The attentional condition being tested is mindful colouring-in for relaxation. Adult art therapy, mindful, and relaxation colouring-in books are now widely available to purchase as a self-help resource.

## Methods/design

### Study aims and objectives

The aim of the study is to establish the feasibility of conducting a definitive RCT of interpersonal art psychotherapy, with a primary focus on implementation and acceptability. The feasibility study will be carried out in four NHS secure hospital sites in England with treatment being provided by trained art psychotherapists who are registered with the UK Health and Care Professions Council (HCPC).

The study objectives for the assessment of feasibility are:

1. Recruitment and consent, such as patients willingness to be randomised and clinicians willingness to recruit their patients into the study.
2. Identifying issues related to seeking informed consent and risks of coercion, including potential for patients to participate in the study believing it will positively or negatively influence their inpatient treatment or detention under the mental health act.
3. Procedures and materials, including suitability of study information, suitability of outcome measures, appraising burden of outcome measures and validated tools and suitability of outcome data collection procedures for maintaining data integrity from multiple study sites.
4. Describing routine care and treatment as usual, identifying characteristics of treatment as usual across multiple sites, levels of high and medium/low security, and individualised patient care pathways.
5. Attrition and acceptability, including rates of attendance for treatment and the acceptability of a novel attentional condition, reasons for non-attendance and/or drop-out, and lack of retention for data collection at the follow-up points.
6. Identifying risks of contamination, such as patients on the waiting list receiving active components of the treatment during routine care and/or in the attentional condition.
7. Treatment fidelity, identifying therapist adherence with the required activity in the treatment manual and piloting treatment fidelity checklist and procedures.

### Design

#### Waiting list control

This multi-centre feasibility study will utilise a parallel-group, participant-randomised design, with participants being allocated to either manualised interpersonal art psychotherapy and treatment as usual, or treatment as usual only while being placed on a waiting list for interpersonal art psychotherapy.

#### Single-case design

Four participants will be separately recruited to take part in single-case multiple-intervention ‘ABACA’ design studies [18] where measures are administered across ‘A’ = baseline (pre-therapy), ‘B’ = first treatment (mindful colouring-in attentional condition), ‘A’ = baseline (wash out period), ‘C’ = second treatment (interpersonal art psychotherapy), ‘A’ = baseline (post-therapy).

#### Client interviews

A brief post-therapy semi-structured interview will be carried out with participants. Questioning will explore helpful and unhelpful aspects of therapy and the participant’s experience of taking part in the study.

#### Treatment fidelity and contamination

Audio recordings from all therapy sessions will be collected. A random sample of 25% of recordings will be blind rated using a therapy checklist by a researcher independent of all other aspects of the study. This assessment will test treatment differentiation (did the providers only deliver the target treatment and not other treatments), treatment competency (did providers maintain the skill set learned in training), and treatment adherence (delivery of the treatment components as intended) [21]. The same procedure will be repeated for audio recordings of the attentional condition to check for presence or absence of components that could constitute contamination from the manualised treatment.

### Intervention

#### Interpersonal art psychotherapy

Interpersonal art psychotherapy has been developed and manualised from practice-based evaluations [13–15]. Directions for the therapist, in terms of style, approach, and techniques, are given in detail in the manual. The interpersonal component of the treatment manual is informed by the core conflictual relationship theme approach [22].

Interpersonal art psychotherapy is delivered in 15 therapy sessions of up to 1 h. Four HCPC registered art psychotherapist (one in each site) have received a 2-day training in the use of the treatment manual. The typical structure of therapy sessions includes the therapist giving an introduction to the session content followed by a directed art activity and a discussion between the therapist and participant.

Including drawing tasks in sessions helps therapists and clients to remember what has been said or what the focus of a session was. Drawings can act as a helpful record that can be referred back to during the course of therapy. In clinical practice it has also been the case that the act of drawing in therapy sessions has prompted some people to remember details of an event they are trying to recall.

Initial therapy sessions focus on helping the patient to identify immediate problems followed by identifying their existing positive coping skills and how they are being used and/or introducing additional coping skills if needed. The patient is then asked to give accounts of their relationships and examples of social interactions. The patient is then asked to think about current or past events and draw pictures of them to aid discussion with the therapist. The content that has been elicited and discussed with the patient in the proceeding sessions is then used to develop or formulate a shared understanding with the patient about reoccurring themes in their accounts of interpersonal interactions that are strongly associated with conflict or aggression. Near the end of therapy, the patient is asked to look ahead and think about themselves in the future including their aspirations or specific personal goals. To end the therapy, a review of the content of all sessions takes place and the patient leaves with a personalised summary and closing letter from the therapist.

The session schedule is as follows: 1 to 3—identifying current problems and coping strategies; 4 to 5—relating to others; 6 to 10—personal life events; 11 to 12—interpersonal themes; 13 to 14—imagining the future; and 15—end of therapy review. The sessions focus on combined components to develop a shared understanding with the client about their dominant interpersonal styles of interacting with others. This includes raising awareness of themes related to the client’s underlying needs or ‘wish’ for particular responses from the people they are interacting with.

#### Attentional condition

Within the single-case design, an attentional condition will be provided. Participants are asked to complete 15 sessions of up to 1 h of mindful colouring-in. During the activity, participants will be given additional instructions by the therapist to breathe slowly and notice what they are doing ‘in the here and now’. Sessions will be delivered by a qualified art psychotherapist and will be equivalent in dose, in terms of number of sessions, to the manualised interpersonal art psychotherapy treatment. Criteria for assessing acceptability of the attentional condition in this study will include the participant’s perception of appropriateness of the intervention for addressing the target problem and willingness to adhere to treatment [23].

### Outcomes

#### Feasibility outcomes

Objective 1, willingness to be randomised and clinician’s willingness to recruit, will be evaluated by assessing the number of eligible patients who were recruited at each site and the number of patients who declined (Additional file 1). For objective 2, issues related to seeking informed consent and potential for coercion will be reported by locally based members of the research team. The same reporting process will be utilised for objective 3 with research assistants reporting feedback from patients at each data collection point on the burden of outcome questionnaires. The level of completion of questionnaires (instrument and item response rates) will be monitored within routine data integrity checks. Objective 4, describing routine care, will be carried out from reviewing individual patient treatment plans. Objective 5, monitoring attrition and acceptability, will be addressed by a combination of recording attendance to treatment and from post-therapy client interview reporting and retention for follow-up data collection. Objective 6, assessing contamination, will be met by crosschecking information collected for objective 5 and by using the treatment fidelity checklist being used to assess objective 7, therapist adherence to the manual, on a sample of audio recordings from the attentional control condition.

#### Primary outcome

Outcome measures will be used to carry out objective 3. The Modified Overt Aggression Scale (MOAS) [19] measures both the frequency (< 10 or > 10 observations) in the previous 7 days and the severity of aggression. It measures four types of aggression: (a) verbal aggression, (b) physical aggression against objects, (c) physical aggression against self, and (d) physical aggression against other people. Agreement between raters for MOAS total scores is (intra-class correlation coefficient (ICC)) of 0.93, verbal aggression (ICC = 0.90), and physical aggression against others (ICC = 0.90) [24].

#### Secondary outcomes

A battery of validated psychological and quality of life measures will be administered at pre-therapy, post-therapy, and follow-up including the Brief Symptom Inventory (BSI) [25], Novaco Anger Scale (NAS) [26], ICEpop CAPability Quality of Life measure for Adults (ICECAP-A V2) [27], and Glasgow Anxiety Scale for people with Intellectual Disability (GAS-ID) [28].

Frequency of routine clinical risk incidents such as periods of seclusion, use of rapid tranquilising medication, and use of prevention and management of violence and aggression techniques will be logged.

A semi-structured client interview will be conducted with participants following the end of treatment. Questions are designed to elicit responses from the participant about helpful and unhelpful aspects of therapy and the suitability and acceptability of treatment with additional questions about participant’s experience of taking part in the study. Thematic analysis [29] will be carried out on all interview transcripts.

Participants included in the single-case design component of the study will complete an additional continuous measure in the form of a daily anger rating scale. This is a visual self-report ‘thermometer style’ anger scale which has been adapted and simplified as recommended by our PPI (Patient and Public Involvement).

#### Study settings

Participants will be recruited from four NHS secure hospital sites in England including three medium/low secure hospitals and one high secure hospital.

##### Inclusion criteria for patients

- An inpatient in a NHS secure hospital having been assessed with IQ of between 55 and 79 (within a range including moderate/mild/borderline intellectual disability). Age 18 to 60 years (within the age range for patients treated in the service) and able to give informed consent. Having a historic and continuing presentation of emotional control difficulties and/or observed aggression or antagonistic behaviour that puts the individual at odds with other people (as identified by the clinical team).
- The patient’s involvement in the study is supported by their responsible clinician and/or multidisciplinary care team.

##### Patient exclusion criteria

- Unable to give informed consent. Having no clinical indicators for psychotherapeutic treatment. Planned discharge from hospital within 12 months of the start of the study.
- Receiving active assessment or treatment, including medication dose titration, for acute psychotic symptoms.

### Randomisation and allocation

Blinding to treatment is not possible in this study. The random allocation sequence was generated using a computer-based statistical software package. During the study, the random allocation sequence will be held by a research assistant who is wholly independent from the recruitment process. Allocation concealment will be in place beyond the first assessment point following which local therapists can request the allocation via email contact with a research assistant on a participant by participant basis. Participants can then be informed if they have been assigned to either interpersonal art psychotherapy or to the waiting list and re-assessed at 4 months by a research assistant.

### Ethics and consent

Independent ethical approval has been granted by a local research ethics committee and the NHS Health Research Authority. The patients taking part in this study will have learning disabilities and be receiving inpatient care. Capacity to consent will be checked in the following ways: the Mental Capacity Act of 2005 will be followed and the patient’s responsible clinician will be advised that capacity to consent to take part in research is in the study inclusion criteria. Should there be any concern raised by the patient, their responsible clinician/care team or a member of the research team regarding capacity to consent an ‘empirical assessment of capacity to consent’ [30] will be carried out by a member of the research team. No further action will be taken if the patient is assessed to lack capacity. Patients will be initially approached by their responsible clinician or a member of their immediate care team to inform them about the study. If the patient would like further information about taking part in the study, this will be provided in written form via the study information sheet. Should patients require this information to be read aloud to them, this will be carried out by care staff who work closely with them and know them well. Patients will be given a minimum of 2 days to decide if they would like to take part in the study after receiving the study information sheet allowing them time to consider the information. Within the study information and consent form, patients will be informed that they can withdraw from the study at any time if they change their mind about participation, without penalty. If patients withdraw during the study, they will be asked if the researchers can include data collected up to that point in the study. Should they wish to proceed, they will be invited to a one-to-one meeting with a member of staff or research assistant and the study information and consent sheet will be reviewed with them and read aloud should they require this. Patients will be invited to have a member of staff who knows them well present at this meeting should they so wish. The voluntary nature of their involvement in this study and being able to withdraw from the study at any point without prejudice to further treatment will be explicitly stated on the study consent form. Recruitment of patients involved in current research or having recently been involved in research is not an exclusion criteria for participation in this study. We will not be asking potential participants to declare if they have recently been involved in research during any stage of the study.

### Confidentiality

All members of the study team are NHS staff and/or contracted to follow NHS trust policies and procedures for confidentiality and information governance in line with good clinical practice. No identifiable information about participants will be used in any report or publication. All personal data from participants will be anonymised using a unique identifier code number.

### Sample size

Twenty patients will be randomly assigned across both treatment allocations. The sample size for this feasibility study is limited due to a number of factors including limitations of time and budget, the intensity of the intervention, and the nature of the settings that it is taking place in. Participants are being recruited from four NHS hospitals in England with low, medium, and high levels of security. Smaller numbers of participants in multiple sites maximises the potential to assess feasibility objectives across a range of complex secure healthcare settings. This multi-site study will assist in future trial planning and inform feasibility from multi-level perspectives regarding issues and occurrences that could inform procedures related to recruitment, treatment acceptability, therapist adherence, and institutional support.

### Single-case design

Four patients (non-randomised) will be recruited for a single-case design component of the study to assess the acceptability of an attentional condition.

### Data management

Data will be collected at each study site including demographic data and current treatment; outcome measures will be collected during baseline, pre-therapy, post-therapy, and at follow-up assessment. Data management procedures will comply with NHS policies on information governance and data protection. Data will be anonymised using a unique participant identifier code and entered onto a password-protected NHS computer. Digital audio data will be wiped from the recording device after being transferred to an NHS password-protected computer. Consent to use an audio digital recording of therapy sessions is included on the consent form. Hard copies of questionnaires and interview transcripts will be stored in a locked filing cabinet in secure offices.

### Statistical methods

Simple descriptive estimation of parameters, for example mean and standard deviation (SD), will be carried out for the outcome measures. Estimation will be carried out using the population as a whole and by group. The numeric measures will be summarised using mean, median, SD, and interquartile range while categorical data will be expressed in terms of the proportion (or percent) of participants within each category and available sample size for each measure will be quoted throughout. Estimates of mean and SD for the measures will be carried out considering each time point separately. Feasibility study objectives will be summarised in the manner of categorical data. Estimates will be used to inform future study design.

### Data monitoring and interim analyses

A data monitoring committee will not be in place. Negative reactions and experiences of participants in the study and/or adverse clinical concerns can be raised by the participant, their responsible clinicians, or members of their immediate care team. It is an inclusion requirement that the participant’s responsible clinician and/or multidisciplinary care team support their involvement in the study and are therefore informed about the nature of the treatment being carried out.

### Harm

Interpersonal art psychotherapy and mindful colouring-in are non-invasive and non-intrusive interventions; however, individual psychological therapies can raise issues of concern for some patients. The main focus of the treatment in this study is to support a reduction in patient’s harmful and anti-social behaviours. To reduce the potential for participants to experience increased levels of distress, both in and following therapy sessions, the treatment will be conducted by qualified art psychotherapists in private/confidential rooms in familiar surroundings with trained members of the patient immediate care team on hand to support them should this be required.

### Auditing

The sponsor can select this study for audit either ‘for cause’ or as part of regular annual audits. Auditing would include a review of the trial master file and/or investigator site file/s.

### Protocol amendments

Notification of both substantial and/or non-substantial protocol amendments will be directed to the responsible Research Ethics Committee for consideration.

### Access to data

Only those in the research team will have access to the data.

### Post-trial care

All patients will continue to receive usual care in the NHS secure setting they are being treated in. Study information forms given to participants include information about where they can raise concerns or complaints related to the study. Study information states that if they have any concerns they can speak to a member of the research team, a member of their immediate care team, or their local NHS patient advice and liaison service (PALS) representative.

### Dissemination

The feasibility study results will be communicated via conference presentations and peer-reviewed publications and via participating NHS Trust and University websites.

## Discussion

This study has been designed to establish feasibility and inform future design of a RCT to evaluate the effectiveness of manualised interpersonal art psychotherapy. Findings will inform the procedures and provide data to calculate the standard deviation of outcome measures and degrees of certainty and estimation of numbers of patients required for a future trial. Additional feasibility objectives will explore therapist adherence to the manual, study, and treatment acceptability and inform the design of a novel attentional control condition.

Potential challenges within this study include clinician’s willingness to approach patients for the study without prior knowledge of the manualised interpersonal art psychotherapy treatment. Strategies being employed to support uptake at sites include providing bespoke information to multidisciplinary teams and, where needed, specific site visits to give presentations to the wider clinical teams.

It is clear that there are a small number of evidence-based psychological treatments for people with learning disabilities [20] and it is important that feasibility work is carried out to maximise the potential for successful completion of RCTs. The study aims and objectives will support a robust assessment of feasibility and inform future trial design.

## Additional file

Additional file 1: Participant Flowchart. (PDF 175 kb)

## Abbreviations

BSI: The Brief Symptom Inventory
GAS: Glasgow Anxiety Scale for people with Intellectual Disability
HCPC: Health and Care Professions Council
HTA: Health Technology Assessment
ICECAP-A: ICEpop CAPability Quality of Life measure for Adults
MOAS: The Modified Overt Aggression Scale
NAS: Novaco Anger Scale
NICE: National Institute for Health and Care Excellence
NIHR: National Institute for Health Research
PALS: Patient advice and liaison service
QoL: Quality of life
RCT: Randomised controlled trial
SD: Standard deviation
