# Supplementary figures and images for: Interpersonal art psychotherapy for the treatment of aggression in people with learning disabilities in secure care: a protocol for a randomised controlled feasibility study
Source: Pilot Feasibility Stud. 2017 Oct 10;3:42. doi: 10.1186/s40814-017-0186-z (PMC5633903; doi:10.1186/s40814-017-0186-z)

Participant Flowchart

Enrolment

Allocation

Follow-up

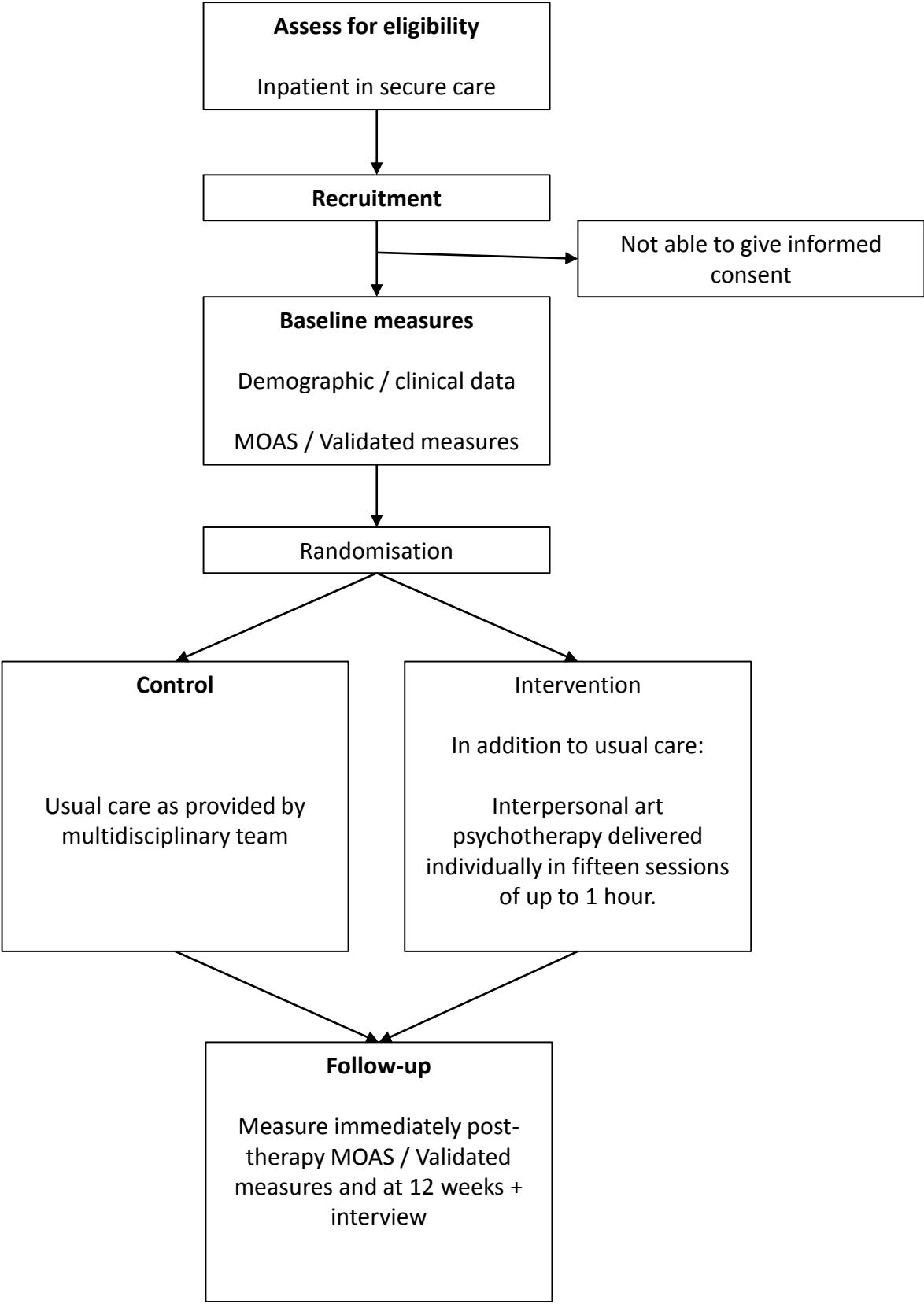

Supplement: Additional file 1: — Participant Flowchart. (PDF 175 kb) [file 40814_2017_186_MOESM1_ESM.pdf]
